# Hypertension Subtypes among Hypertensive Patients in Ibadan

**DOI:** 10.1155/2014/295916

**Published:** 2014-10-19

**Authors:** Abiodun M. Adeoye, Adewole Adebiyi, Bamidele O. Tayo, Babatunde L. Salako, Adesola Ogunniyi, Richard S. Cooper

**Affiliations:** ^1^Department of Medicine, University of Ibadan, Ibadan, Nigeria; ^2^Department of Public Health Sciences, Loyola University Chicago Stritch School of Medicine, Maywood, IL 60153, USA

## Abstract

*Background.* Certain hypertension subtypes have been shown to increase the risk for cardiovascular morbidity and mortality and may be related to specific underlying genetic determinants. Inappropriate characterization of subtypes of hypertension makes efforts at elucidating the genetic contributions to the etiology of hypertension largely vapid. We report the hypertension subtypes among patients with hypertension from South-Western Nigeria. *Methods.* A total of 1858 subjects comprising 76% female, hypertensive, aged 18 and above were recruited into the study from two centers in Ibadan, Nigeria. Hypertension was identified using JNCVII definition and was further grouped into four subtypes: controlled hypertension (CH), isolated systolic hypertension (ISH), isolated diastolic hypertension (IDH), and systolic-diastolic hypertension (SDH). *Results.* Systolic-diastolic hypertension was the most prevalent. Whereas SDH (77.6% versus 73.5%) and IDH (4.9% versus 4.7%) were more prevalent among females, ISH (10.1% versus 6.2%) was higher among males (*P* = 0.048). Female subjects were more obese (*P* < 0.0001) and SDH was prevalent among the obese group. *Conclusion.* Gender and obesity significantly influenced the distribution of the hypertension subtypes. Characterization of hypertension by subtypes in genetic association studies could lead to identification of previously unknown genetic variants involved in the etiology of hypertension. Large-scale studies among various ethnic groups may be needed to confirm these observations.

## 1. Introduction

Cardiovascular disease is the world's number one killer. World Heart Federation statistics reveal that cardiovascular diseases account for 17.3 million deaths per year, and by 2030 this is expected to rise to 23 million. Hypertension remains a worldwide phenomenon being a major component of cardiovascular diseases with life-time cumulative incidence approaching 50% in many populations [1]. Mass migration from rural to peri-urban and urban areas with improved industrialization and adoption of western diets and lifestyle changes have led to steady increase in incidence of hypertension in Africa [2, 3]. In sub-Sahara alone, about 10–20 million people have hypertension with various degrees of target organ damages [4]. Based on the data available, the African Union has identified hypertension as one of its challenges after AIDS [3, 5].

In Nigeria, the prevalence of hypertension is on the increase among both the rural and urban settlers with resultant rising trends of sudden cardiac death [6–9]. Currently about 36 million Nigerians are estimated to have hypertension and its associated complications [9]. Despite all the advances in the study and management of hypertension, the control remains very poor. Salako et al. [10] found only 25.4% of subjects studied in a clinical setting had both systolic blood pressure (SBP) and diastolic blood pressure (DBP) controlled. Uncontrolled hypertension accounts for substantial proportion of cardiovascular deaths and morbidity resulting from stroke, heart failure, acute myocardial infarction, and kidney failure [11–13]

Prevention of hypertension and its control can markedly reduce cardiovascular morbidity and mortality; however the multifactorial aetiopathophysiologic mechanism of hypertension makes control difficult. A complex relationship within the environment and genetics accounts for the etiology of this disease. While the environmental factors such as obesity, diet (especially high sodium, low potassium, and excess energy intake), stress, and physical inactivity are easily elucidated, the genetic determinants of hypertension remain obscure. Heterogeneity within patient subsets and attempts to combine all hypertensives together in the search of genes has made genetic study of hypertension highly challenging and mostly difficult. It is possible that hypertension is due to multiple distinct genes that can be studied better by subdividing hypertensive subtypes and this approach may help elucidate the genetics and pathophysiology of hypertension. The search for homogeneous hypertension subtypes has in the recent years widened the scope of our understanding of monogenic Mendelian hypertension [14]. It was shown that primary hypertension with hypokalemia has different pathophysiologic mechanism than those without hypokalemia. Also Jiménez et al. [15] described an association between predominantly diastolic hypertension (PDH) subtypes and the angiotensin-converting enzyme DD polymorphism in a small population of untreated patients with PDH. Most of the studies being undertaken to define the genetic influences have been in western societies where high levels of exposure to environmental risk factors prevail, especially obesity and excess sodium intake [16, 17]. To broaden the perspective on the subtypes of this condition we report a case series of subjects with hypertension at the University College Hospital and Adeoyo State Hospital Ibadan, Nigeria.

## 2. Materials and Methods

### 2.1. Study Design and Population

This cross-sectional descriptive study was conducted at the Medical Outpatient clinics of University College Hospital and Adeoyo State Hospital Ibadan, a secondary healthcare center, Oyo State, South West Nigeria. The two hospitals serve as referral centers for primary health care centers in Ibadan. Ibadan is the capital city of Oyo State in the south-western area of Nigeria and has a population of 3.6 million, while Oyo State has 5.6 million people according to the National Population census 2007. The Yoruba ethnic group is the major tribe in Ibadan city, while other major Nigerian ethnic groups like Igbo and Hausa are fairly represented. Christianity, Islam, and traditional religions are widely practiced in Ibadan. The city has a tropical wet and dry climate with a lengthy wet season and relatively constant temperatures throughout the course of the year. There are two peaks for rainfall, June and September. The mean maximum temperature is 26.46°C, minimum 21.42°C, and the relative humidity is 74.55%.

A total of 1858 hypertensive subjects, aged 18 years and above, of Yoruba tribe comprising 1411 females and 447 males were recruited into the study over two and half years between June 2009 and December 2011. Written informed consent was obtained from all participants. Only consenting participants of Yoruba tribe were included in the study. Subjects with fasting plasma glucose of greater than 126 mg/dL and plasma creatinine of greater than 1.5 mg/dL and those that refused consent were excluded from the study. The research protocol was approved by the joint Ethics Committee of the University College Hospital/University of Ibadan, Nigeria, and by the Institutional Review Board at Loyola University Medical Center, Maywood, IL, USA.

### 2.2. Data Collection

All measurements were conducted by one trained physician and two nurses between 8:00 am and 12:00 pm at Adeoyo State Hospital and 2 pm and 6 pm at medical outpatient, University College Hospital, Ibadan respectively. Blood pressure (BP) was measured using a standard Omron (HEM711DLX) blood pressure apparatus on the left arm after 5-minute rest using a cuff of appropriate size with the subject in the sitting position. Three BP measurements were obtained with a minimum interval of one minute and mean values were used in the analysis. Anthropometric measurements including height, weight, and waist and hip circumferences were obtained. Height was measured without shoes to the nearest centimeter using a ruler attached to the wall, while weight was measured to the nearest 0.1 kg on an electronic scale with the subject wearing light outdoor clothing and no shoes. Waist circumference was measured at the narrowest part of the participant's torso (or the minimum circumference between the rib cage and the iliac crest) [18] using an anthropometric measuring tape. The measurement was taken at the end of expiration. We measured waist circumference, recorded to the nearest tenth of a centimeter, 3 times and used the average of the 3 measurements.

Hypertension was defined as SBP ≥ 140 mmHg and/or DBP ≥ 90 mmHg or being on pharmacological treatment for hypertension. Hypertension subsubtypes were defined as follows: controlled hypertension (CH) if on antihypertensive medication, SBP < 140 and DBP < 90; isolated systolic hypertension (ISH) if SBP ≥ 140 and DBP < 90; isolated diastolic hypertension if SBP < 140 and DBP ≥ 90; systo-diastolic hypertension (SDH) if SBP ≥ 140 and DBP ≥ 90; and predominantly diastolic hypertension (PDH) group as subjects having a pulse pressure (PP) to DBP ratio < 0.45.

Obesity was classified based on body mass index (BMI) in kg/m^2^ as normal (<25 and >20), overweight (>25 and <30), obesity (>30 and <35), and severe obesity (≥35). Abdominal obesity was defined as waist circumference of greater than or equal to 102 cm in men and greater than or equal to 88 cm in women.

### 2.3. Statistical Analysis

Data was analyzed using the Statistical Package for Social Sciences (SPSS Inc, Chicago, IL) version 15. Results were expressed as either mean values (±standard deviation) or proportions. Comparison for statistical significance was by independent Student's *t*-test for continuous variables or chi-square for categorical variables. One way analysis of variance (ANOVA) with Bonferroni's post hoc method was used to compare the demographic and BP indices among the various BP subtype groups. The level of significance was set at *P* ≤ 0.05.

## 3. Results

**Figure 1:**
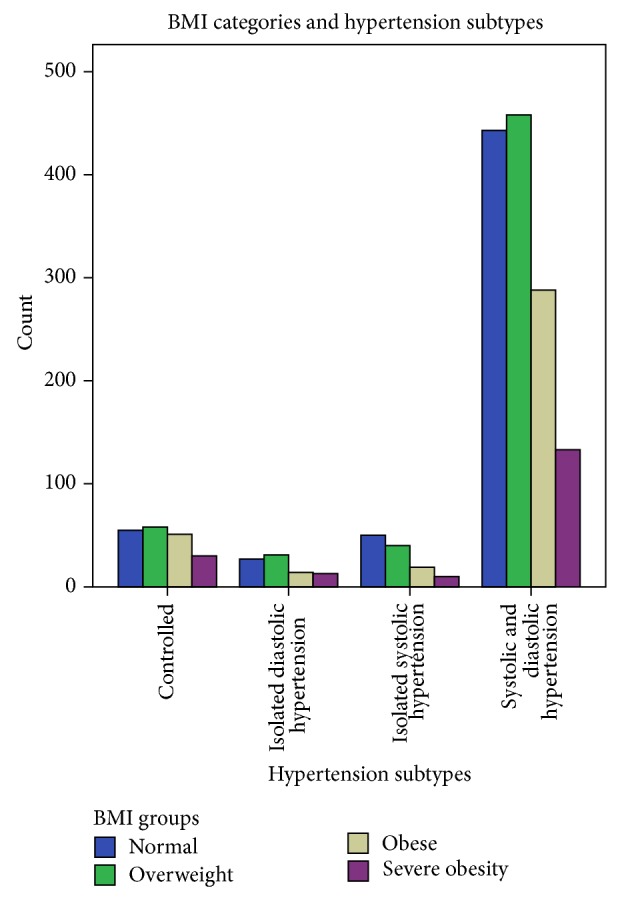
BMI categories and hypertension subtypes.

**Figure 2:**
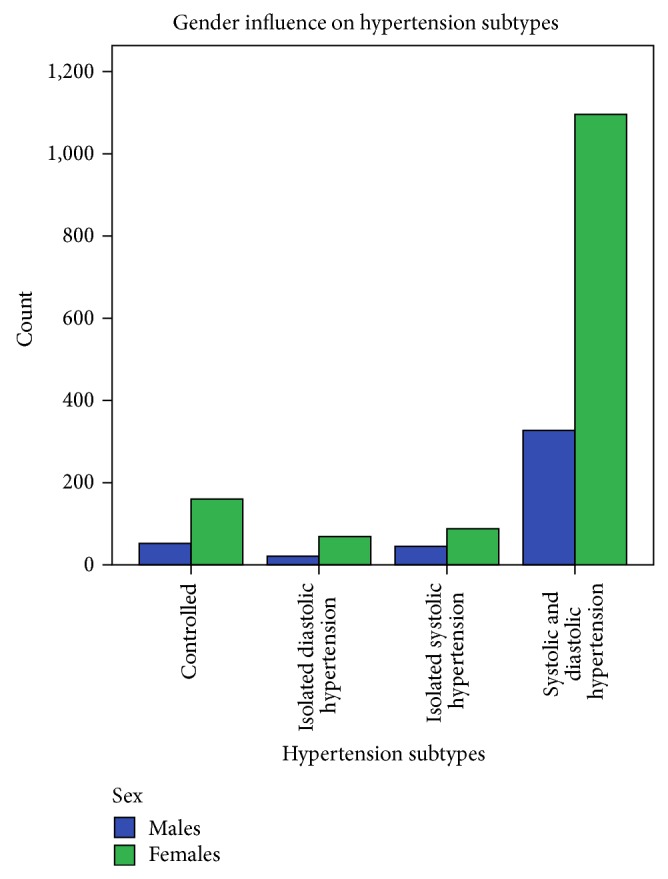
Gender influence on hypertension subtypes.

A total of 1858 hypertensive subjects (mean age 49 ± 9years) comprising 1411 females and 443 male participants were recruited into the study. The characteristics of the study population classified by gender are as shown in Table 1. Females were significantly older, shorter, and heavier and had greater arm circumference and increased heart rates when compared with males. Blood pressure parameters were comparable among females and males. Anthropometric measurements showed that 587 (34.1%) were overweight, 372 (21.6%) obese, and 186 (10.8%) severely obese. Compared with males, female subjects were significantly more obese (*P* < 0.0001). Similarly 51.6% of the study population had abdominal obesity with female preponderance (*P* < 0.0001). Also as seen in Figure 1, there was significant effect of obesity on the distribution of hypertension subtypes. As shown in Figure 2, SDH (77.6% versus 73.5%) and IDH (4.9% versus 4.7%) are more prevalent among females compared with males, whereas the prevalence of ISH (10.1% versus 6.2%) and CH (11.7% versus 11.3%) was higher among males.  Table 2 showed age group and gender relations of hypertension subsubtypes. While there was significant gender effect on the frequency of blood pressure subgroup, the age group did not affect the blood pressure subtypes. The frequency of the different hypertension subtypes among the 1858 hypertensive subjects was as follows: controlled hypertension 11.4%, isolated diastolic hypertension (IDH) 4.8%, isolated systolic hypertension (ISH) 7.2%, and systolic and diastolic hypertension (SDH) 76.6%. Predominantly diastolic hypertension (PDH) was observed in 329 (17.7%) of the entire study population, 56 (26.4%) of CH, 72 (80%) of IDH, and 201 (14.1%) of SDH. We observed significant differences between hypertension subtype groups in essentially all the physiologic and anthropometric parameters (Table 3). SDH (77.6% versus 73.5%) and IDH (4.9% versus 4.7%) are more prevalent among females compared with males, whereas the prevalence of ISH (10.1% versus 6.2%) and CH (11.7% versus 11.3%) was higher among males.

## 4. Discussion

This study shows that hypertension is a phenotype consisting of heterogeneous subtypes. The pooling of hypertension patients without consideration for the heterogeneous nature of hypertension subtype in genetic association mapping for hypertension may have contributed to the limited success in identification of genetic variants involved in the etiology of hypertension to date.

Interestingly, studies have shown that the frequencies of various hypertension subtypes depend on the age of the cohorts studied. While some found IDH to be more prevalent among young adults others found it more prevalent among the elderly [19–23]. In the present study, subjects with IDH accounted for 4.8% of the total population and were significantly younger than those of the other groups. A report from China contradicts an early claim that linked IDH blood pressure profile with low cardiovascular risk [24]. It was demonstrated in that study that although less than ISH and SDH, patients with IDH had higher rates of cardiovascular diseases than normotensive individuals [19, 25]. Current findings of prevalent IDH among young cohort as shown in this study require increased research interest in this group of people to prevent further hypertension associated morbidity and mortality.

Isolated systolic hypertension (ISH) is a common “pulse pressure phenotype” that has been associated with increased cardiovascular risk. In Framingham's study [26], ISH was more common among the elderly. It was shown that, among the young adults, ISH was more likely to evolve from high normal or normal blood pressure but in the elderly it most likely emanates from systo-diastolic hypertension (SDH) and IDH making two distinct types of isolated systolic hypertension. These discrepancies might suggest different genetic influence for each type of ISH. Similarly in this study, ISH was more prevalent among the older subjects and also more prevalent than IDH, but the specific categories of ISH subtypes were not studied

The gender bias in favour of females in this particular study may be explained by the nature of the community where health seeking is considered as a feminine behavior until an illness becomes severe. In this study there was a clear evidence of gender influence on the risk factors associated with hypertension. Women were significantly older, shorter, and heavier and had greater arm circumference and heart rates when compared with men. This is similar to study by Ejim et al. [8] who found hypertensive women in the Eastern Nigeria community to be heavier, taller, and older than their male counterparts. Elevated heart rate has been shown to be a risk factor for cardiovascular morbidity and mortality especially among the hypertensive [27]. Other studies also showed that elevated heart rate potentiates the risk of metabolic disturbances, diabetes, and atherosclerosis and coronary artery diseases [28]. Significantly higher heart rates among female as demonstrated in this study might potentiate the increased cardiovascular risk in them.

Some studies showed that patients with IDH have high prevalence of metabolic syndrome or increased body mass index [29, 30]. In our study the IDH subtypes had increased body mass index compared to the finding of Jiménez et al. [15]. From the foregoing, the IDH subsets in our study require more attention in terms of management of hypertension and prevention of cardiovascular morbidity and mortality.

Orias et al. [31] suggested that predominantly diastolic hypertension (PDH) describing SDH subsets with narrow pulse pressure tends to have similar hemodynamic patterns and are more homogenous. Also Jiménez and colleagues [15] described a link between PDH and angiotensin converting enzyme (ACE) polymorphisms in a small number of untreated hypertensives. Similar observation was reported in the Framingham cohort, which showed strong association between ACE genotype and diastolic blood pressure among men [26]. Using Blank and associates [20] definition, the prevalence of PDH in this study was 17.7% of general population accounting for 26.4% among CH, 80% of IDH, and 14.1% of SDH. PDH and IDH have been shown to have similar physiology which if it applies cumulatively makes the prevalence of IDH in this study almost 20% of the subject population. Some studies demonstrated the prevalence of IDH to be as high as 23%. When patients with PDH are added to the group, IDH may account for 30%–40% of subjects with essential hypertension [29]. Although not conclusive it is tempting to suggest from the study that 20–25% of our studypopulation have similar haemodynamic and genetic makeup. This may suggest an association between this group and ACE genotype.

This study has shown that the heterogeneity of hypertension may also determine the degree of blood pressure control among subjects with hypertension. Gender and obesity significantly influenced the distribution of the hypertension subtypes. Prevention or control of hypertension would be better if the various subtypes are well understood. From this study, reasons for the low frequency of controlled hypertension might just not be due to nonavailability of drugs or patients' poor drug adherence but also likely resulting from the varying prevalence and characteristic of hypertension subtypes as elucidated in this work.

Our study has the following limitations. The findings of this study's results may not be generalizable to the whole population because individuals attending hospital may have other comorbidities that were not taken into cognizance in this study. There is the possibility of sessional variation bias in population survey of blood pressure. However, Ibadan city has a tropical wet and dry climate with a lengthy wet season and relatively constant temperatures throughout the course of the year; we tend to believe that seasonal variation could not have significantly affected our findings in this study. Also, since the subjects were hypertensives on drug therapy, these could have introduced some misclassification into the subtype determination.

## 5. Conclusion

We have characterized the heterogeneous nature of hypertension with the predominantly Yoruba-speaking population of southwest Nigeria. As part of effort to identify genetic variants involved in the etiology of hypertension, the different hypertension subtypes may warrant individual consideration. Future research endeavors might focus on the young adults with isolated diastolic hypertension to prevent potential early cardiovascular morbidity and mortality. Larger studies in multiple populations may be needed to provide further insight into this.

## Figures and Tables

**Table 1:** Basic characteristics of the population.

Variable	Female (*n* = 1411)	Male (*n* = 447)	Total (*n* = 1858)	*P* value
Age (yrs)	48.9 (8.29)	47.2 (10.58)	48.5 (8.92)	<0.002^*^
Weight (kg)	70.7 (15.20)	71.2 (14.15)	70.9 (15.66)	0.521
Height (m)	1.6 (0.07)	1.7 (0.07)	1.61 (8.09)	<0.001^*^
Arm circumference (cm)	29.1 (4.14)	27.8 (3.12)	28.8 (4.01)	<0.001^*^
Heart rate	89.7 (16.42)	85.9 (15.85)	89.1 (18.61)	<0.001^*^
Systolic blood pressure (mmHg)	161.6 (25.50)	161.2 (24.15)	161.8 (26.99)	0.766
Diastolic blood pressure (mmHg)	101.4 (14.07)	100.6 (15.23)	101.4 (15.80)	0.338
Body mass index (kg/m^2^)	28.1 (5.80)	25.2 (4.80)	29.13 (7.27)	<0.001^*^
BMI groups				<0.001^*^
Normal	376 (28.5)	199 (49.8)	575 (33.4)	
Overweight	447 (33.9)	140 (35.0)	587 (34.1)	
Obese	320 (24.2)	52 (13.0)	372 (21.6)	
Severe obesity	177 (13.4)	9 (2.3)	186 (10.8)	
Waist-to-height ratio	0.708 (0.16)	0.712 (0.14)	0.709 (8.10)	0.635
Abdominal obesity	496 (35.1)	42 (9.4)	969 (51.6)	<0.001^*^

^*^Statistically significant.

**Table 2:** Age group and gender relations of hypertension subtypes.

	Controlled hypertension (*n* = 212)	Isolated diastolic hypertension (*n* = 90)	Isolated systolic hypertension (*n* = 133)	Systolic and diastolic hypertension (*n* = 1423)	*P* value
Gender					<0.048
Females	160 (11.3)	69 (4.9)	88 (6.2)	1096 (77.6)	
Males	52 (11.7)	21 (4.7)	45 (10.1)	327 (73.5)	
Age group					0.254
Young (≤39 yrs)	14 (6.6)	11 (12.2)	13 (9.8)	107 (7.5)	
Middle age (40–59 yrs)	185 (87.3)	76 (84.4)	107 (80.5)	1201 (84.4)	
Elderly (≥60 yrs)	13 (6.1)	3 (3.3)	13 (9.8)	115 (8.1)	

**Table 3:** Comparisons of demographic and BP indices in the hypertension subtypes.

Parameters (means)	Controlled	Isolated diastolic hypertension	Isolated systolic hypertension	Systolic and diastolic hypertension	*P* value
Age	48.80 (8.52)^a^	45.40 (8.78)^b^	50.14 (9.56)^a^	48.51 (8.88)^a^	<0.001^*^
SBP	122.98 (11.46)^a^	133.15 (5.80)^b^	155.33 (12.56)^c^	169.98 (28.83)^d^	<0.001^*^
DBP	80.14 (6.64)^a^	97.18 (9.47)^b^	84.58 (4.90)^c^	106.41 (13.85)^d^	<0.001^*^
Waist	95.02 (13.53)^c^	93.41 (12.23)^b,c^	90.08 (11.84)^a^	90.78 (12.48)^a,b^	<0.001^*^
Pulse	83.47 (15.22)^a^	92.40 (16.09)^c^	87.70 (25.11)^a^	89.83 (18.36)^b,c^	<0.001^*^
Waist-to-height ratio	0.74 (0.16)^b^	0.72 (0.17)^a,b^	0.69 (0.15)^a^	0.71 (0.16)^a^	<0.003^*^
BMI	28.14 (6.23)^b^	27.89 (5.98)^b^	25.97 (5.39)^a^	27.46 (6.24)^b^	<0.014^*^
PDH ratio^**^	0.54 (0.15)^a^	0.38 (0.10)^b^	0.84 (0.18)^c^	0.60 (0.19)^d^	<0.001^*^
Weight	74.35 (15.94)^b^	72.06 (16.85)^a,b^	68.63 (14.63)^a^	70.56 (15.57)^a^	<0.003^*^
Height	162.85 (7.41)^b^	160.56 (9.63)^a^	162.63 (8.02)^b^	160.50 (8.03)^a^	<0.001^*^

^a,b,c,d^Means with the same superscript are not different at *P* < 0.05.

^*^Statistically significant.

^**^PDH ratio = pulse pressure/diastolic pressure.
